# Cartilage Oligomeric Matrix Protein Promotes Radiation Resistance in Non-Small Cell Lung Cancer In Vitro

**DOI:** 10.3390/ijms26062465

**Published:** 2025-03-10

**Authors:** Kaitlyn E. Reno, Alicia Costa-Terryll, Sun H. Park, Ryan T. Hughes, Michael K. Farris, Fei Xing, Jeffrey S. Willey

**Affiliations:** 1 Department of Radiation Oncology, Wake Forest University School of Medicine, 1 Medical Center Blvd, Winston Salem, NC 27157, USA; kreno@wakehealth.edu (K.E.R.); acostate@wakehealth.edu (A.C.-T.); sun.h.park@wakehealth.edu (S.H.P.); ryhughes@wakehealth.edu (R.T.H.); mfarris@wakehealth.edu (M.K.F.); 2 Department of Cancer Biology, Wake Forest University School of Medicine, 1 Medical Center Blvd, Winston Salem, NC 27157, USA; fxing@wakehealth.edu

**Keywords:** cartilage oligomeric matrix protein, non-small cell lung cancer, radiation resistance, proliferation, invasion

## Abstract

Cartilage oligomeric matrix protein (COMP) is an extracellular matrix protein that has recently been associated with worse patient outcomes in breast, prostate, colorectal and hepatocellular cancers. This study aimed to determine whether COMP was also associated with increased progression and resistance to radiation in non-small cell lung cancer (NSCLC). The proliferation, migration, invasion and cell viability of wild-type and COMP overexpressing NSCLC cell lines were assessed when treated with exogenous COMP, with or without radiation. In addition, these cells were treated with inhibitors of downstream signaling intermediates of COMP. Proteomics were performed on the A549 cell line treated with COMP, radiation and inhibitors. NSCLC cells treated with COMP or overexpressing COMP had greater proliferation, migration, invasion and viability when irradiated compared to non-overexpressed cells treated with radiation alone, but this effect was reversed when treated with Src or PI3k inhibitors. The NCI-H1437 cell line exhibited a decrease in proliferation when treated with exogenous COMP, however COMP overexpression mitigated the radiation-induced reduction. Proteomics analyses indicate that COMP promotes oxidative phosphorylation and drug resistance pathways. Therefore, COMP overexpression and treatment with exogenous COMP appears to protect NSCLC cells against radiation in vitro, however treatment with inhibitors reverses COMP-mediated protection and progression.

## 1. Introduction

Lung cancer is the second most common cancer in men and women and is the cancer with the greatest number of deaths [1]. Treatment resistance, particularly radiation resistance also remains an issue in lung cancer and is thought to be a contributor to the low survival rate [2,3]. The PI3k/AKT pathway is linked to radiation resistance through the repair of double stranded DNA breaks [4] by preventing apoptosis in the tumor cell [5]. Cartilage oligomeric matrix protein (COMP) is an extracellular matrix protein that binds to the cell surface and activates the PI3k/AKT pathway; thus it is possible COMP expression may be directly associated with radiation resistance in non-small cell lung cancer (NSCLC) [6,7,8].

COMP is a soluble extracellular matrix (ECM) glycoprotein predominantly identified in cartilage, but also present in fibroblasts, vascular smooth cells, myofibroblasts, and tendons [9]. COMP is a member of the thrombospondin family of proteins and is important in cell responses to growth factors and cytokines, as well as matrix assembly [10,11]. When COMP is secreted, it binds to several receptor proteins on the cell surface, including CD36, CD47, and a_v_β_3_ and a_v_β_5_ integrins [6,7,12,13]. These cell surface receptors then activate the Src and PI3k/AKT pathways which also have implications in driving cancer cell proliferation, invasion and metastasis [6,7]. Thus, inhibiting COMP is a promising target for cancer treatments, particularly as COMP has been found to be highly expressed in and causes increased incidence of metastasis and proliferation of multiple cancer types including, breast, prostate, and colorectal cancers [6,12,14,15,16,17,18,19]. Additionally, COMP expression is associated with increased tumor invasiveness through integrin binding [12]; inhibiting a_v_β_3_ and a_v_β_5_ receptors using cilengitide in DU145 cells treated with COMP reduced COMP-mediated integrin activation of the FAK/Src/AKT pathway [20], lowering invasion [12]. Moreover, hepatocellular carcinoma (HCC) cells treated with recombinant COMP enhanced migration and invasion which was found to be related with the PI3k/AKT pathway through the CD36 receptor, a known receptor for the thrombospondin family [6,7,8]. Activated CD36 causes increased lung adenocarcinoma cell proliferation and metastasis [21]. COMP has also been found to be highly expressed in NSCLC cells that contained the EGFR^L858R^ and TP53 mutations [22]. COMP expression in advanced NSCLC tumors from patients receiving first-line pembrolizumab was found to be negatively associated with progression-free-survival (PFS) [23]. While COMP expression in patients with NSCLC was directly associated with poor PFS, this association was in the context of treatment with pembrolizumab only, and not the current common treatment strategies for advanced NSCLC as that was not the aim of the study. Thus, COMP expression is directly associated with elevated proliferation, metastatic potential in NSCLC, and poor patient outcomes in other cancer types.

We hypothesized that increased levels of COMP may promote NSCLC cell proliferation, invasion, and radiation resistance. The purpose of our studies was to evaluate whether either exogenous COMP or COMP-overexpression promoted NSCLC advancement and radiation resistance.

## 2. Results

### 2.1. The Effect of Exogenous COMP on Cell Proliferation and Migration

**Figure 1:**
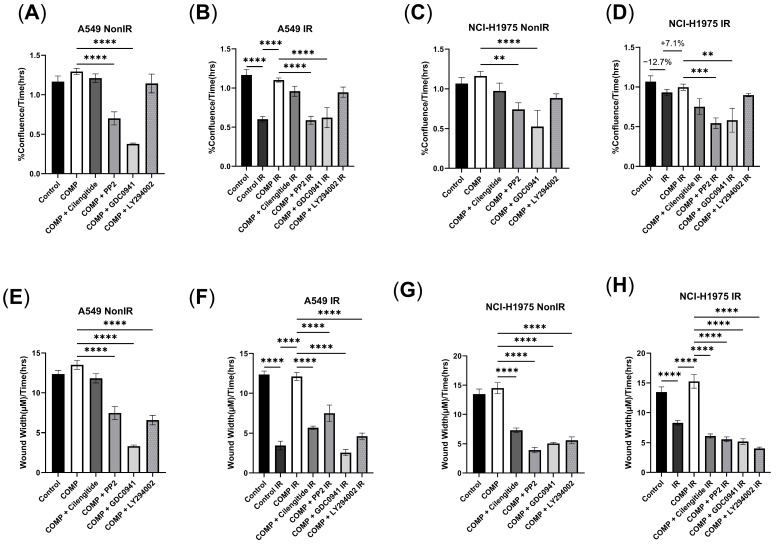
Exogenous COMP promotes a radiation resistant phenotype in NSCLC cells. (**A**) Proliferation rates measured in %confluence/h of non-irradiated A549 cells treated with COMP ± an indirect inhibitor of COMP, either: cilengitide, PP2, GDC0941, or LY294002. (**B**) Proliferation rates of irradiated A549 cells treated with COMP ± an indirect inhibitor of COMP. (**C**) Proliferation rates of non-irradiated NCI-H1975 cells treated with COMP ± an indirect inhibitor of COMP. (**D**) Proliferation rates of irradiated NCI-H1975 cells treated with COMP ± an indirect inhibitor of COMP. (**E**) Migration rates measured in wound width (μm)/time (h) of non-irradiated A549 cells treated with COMP ± an indirect inhibitor of COMP. (**F**) Migration rates of irradiated A549 cells treated with COMP ± an indirect inhibitor of COMP. (**G**) Migration rates of non-irradiated NCI-H1975 cells treated with COMP ± an indirect inhibitor of COMP. (**H**) Migration rates of irradiated NCI-H1975 cells treated with COMP ± an indirect inhibitor of COMP. Statistical analysis done by one-way ANOVA followed by Tukey’s post-hoc test. Error bars represent mean ± SEM (** *p* < 0.01, *** *p* < 0.001, **** *p* < 0.0001).

The proliferation rate for the A549 cell line decreased after 2Gy (−48.6% Control IR vs. Control *p* < 0.0001) (Figure 1B); likewise, the NCI-H1975 cell line exhibited a similar trend (−12.7% IR vs. Control) (Figure 1D). When exogenous COMP was delivered together with radiation, A549 cell proliferation increased vs. irradiated cells that had not been treated with exogenous COMP (+83.5% COMP IR vs. Control IR, *p* < 0.0001) (Figure 1B), with similar responses in NCI-H1975 cells (+7.1% COMP IR vs. Control IR) (Figure 1D). The A549 and NCI-H1975 cell lines exhibited lower migration rates when exposed to 2Gy (*p* < 0.0001) (Figure 1F,H); however, COMP treatment prevented this reduction of migration in both cell lines (*p* < 0.0001) (Figure 1F,H).

COMP signaling intermediates were targeted to further identify if COMP treatment could affect radiation responses in NSCLC cell lines, specifically with inhibitors against integrins, Src and PI3k signaling. COMP treatment + the integrin inhibitor, cilengitide, did not affect proliferation in either cell line (Figure 1A–D). While COMP treatment preserved migration rates for irradiated A549 and NCI-H1975 cells, migration was reduced in both cells lines after irradiation with cilengitide + exogenous COMP treatment (Figure 1F,H). Likewise, while exogenous COMP treatment preserved proliferation after 2Gy in both cell lines, proliferation was reduced after 2Gy when treated with the Src inhibitor, PP2 + COMP (Figure 1B,D). PP2 with exogenous COMP treatment decreased migration vs. COMP treatment and 2Gy in non-irradiated and irradiated A549 and NCI-H1975 cell lines (*p* < 0.0001) (Figure 1E–H). GDC0941, a pan-PI3K inhibitor, with exogenous COMP treatment significantly decreased proliferation compared to COMP treatment alone in non-irradiated and irradiated A549 (*p* < 0.0001) and NCI-H1975 cell lines (*p* < 0.01) (Figure 1A–D). GDC0941 treatment with exogenous COMP decreased migration rates compared to COMP treatment alone in both irradiated and non-irradiated A549 and NCI-H1975 cell lines (*p* < 0.0001) (Figure 1E–H). LY294002, a PI3K inhibitor, did not affect proliferation rates in both cell lines, whether non-irradiated or irradiated (Figure 1A–D). However, while COMP preserved migration rates after 2Gy in both cell lines, LY294002 treatment with exogenous COMP resulted in lowered migration rates in both A549 (*p* < 0.0001) and NCI-H1975 cells (*p* < 0.0001) (Figure 1E–H).

### 2.2. Validation of COMP Overexpression

COMP overexpression was verified using RT-qPCR. The fold change in the COMP overexpressed cells was significantly higher than in the mock-transduced cells for both the A549 and NCI-H1437 cell lines (*p* < 0.001 and *p* < 0.05, respectively) (Figure S1A,B).

### 2.3. COMP Overexpression in the A549 Cell Line and the Response to Radiation with/Without COMP Signaling Inhibitors

**Figure 2:**
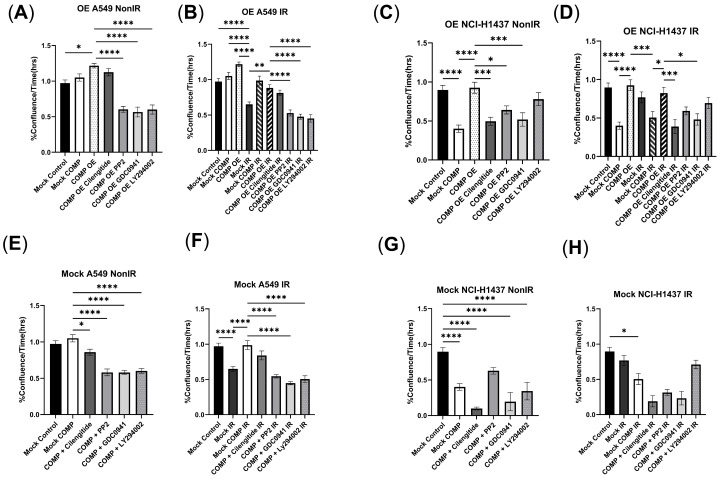
COMP overexpression in NSCLC prevents radiation-induced reduction of proliferation. (**A**) Proliferation rates measured in %confluence/h of non-irradiated mock-transduced or COMP-overexpressed A549 cells treated with either PBS (control), cilengitide, PP2, GDC0941, or LY294002. (**B**) Proliferation rates of irradiated mock-transduced or COMP-overexpressed A549 cells treated with PBS, cilengitide, PP2, GDC0941, or LY294002. (**C**) Proliferation rates of non-irradiated mock-transduced or COMP-overexpressed NCI-H1437 cells treated with either PBS (control), cilengitide, PP2, GDC0941, or LY294002. (**D**) Proliferation rates of irradiated mock-transduced or COMP-overexpressed NCI-H1437 cells treated with either PBS, cilengitide, PP2, GDC0941, or LY294002. (**E**) Proliferation rates measured in %confluence/h of non-irradiated mock-transduced A549 cells treated with PBS or COMP and either no additional treatment, cilengitide, PP2, GDC0941, or LY294002. (**F**) Proliferation rates of irradiated mock-transduced A549 cells treated with PBS or COMP and either no additional treatment, cilengitide, PP2, GDC0941, or LY294002. (**G**) Proliferation rates of non-irradiated mock-transduced NCI-H1437 cells treated with PBS or COMP and either no additional treatment, cilengitide, PP2, GDC0941, or LY294002. (**H**) Proliferation rates of irradiated mock-transduced NCI-H1437 cells treated with PBS or COMP and either no additional treatment, cilengitide, PP2, GDC0941, or LY294002. Statistical analysis done by one-way ANOVA followed by Tukey’s post-hoc test. Error bars represent mean ± SEM (* *p* < 0.05, ** *p* < 0.01, *** *p* < 0.001, **** *p* < 0.0001).

In non-irradiated cells, overexpression of COMP (COMP OE) increased proliferation rates vs. the mock-transduced (Mock) A549 control cell line (*p* < 0.05). Reduced proliferation was observed after 2Gy in the Mock A549 cells vs. non-irradiated Mock A549 cells (*p* < 0.0001) (Figure 2B). However, 2Gy did not reduce proliferation in COMP OE cells vs. the irradiated Mock A549 (*p* < 0.01) (Figure 2B). Likewise, 2Gy did not reduce proliferation in Mock A549 cells that were treated with exogenous COMP (Figure 2B).

The COMP signaling inhibitors cilengitide, PP2, GDC0941, and LY294002 were tested to identify the effects of radiation on proliferation and migration of COMP OE cells. Cilengitide treatment did not affect the 2Gy-induced reduction in proliferation in COMP OE cells (Figure 2B). However, PP2 and GDC0941 treatment resulted in reduced proliferation after 2Gy in the COMP OE cells (*p* < 0.0001), as did LY294002 treatment (*p* < 0.0001) (Figure 2B).

**Figure 3:**
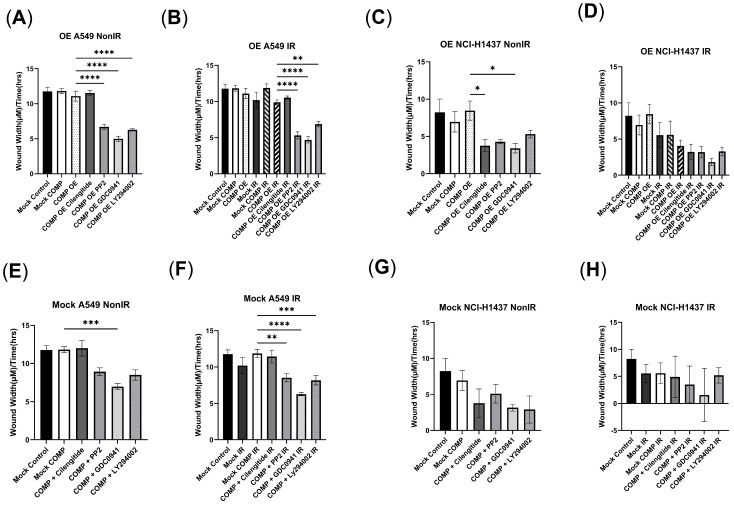
Treatment of COMP overexpressed NSCLC cells with inhibitors reduces migration. (**A**) Migration rates measured in wound width (μm)/time (h) of non-irradiated mock-transduced or COMP-overexpressed A549 cells treated with either PBS (control), cilengitide, PP2, GDC0941, or LY294002. (**B**) Migration rates of irradiated mock-transduced or COMP-overexpressed A549 cells treated with either PBS, cilengitide, PP2, GDC0941, or LY294002. (**C**) Migration rates of non-irradiated mock-transduced or COMP-overexpressed NCI-H1437 cells treated with either PBS (control), cilengitide, PP2, GDC0941, or LY294002. (**D**) Migration rates of irradiated mock-transduced or COMP-overexpressed NCI-H1437 cells treated with either PBS, cilengitide, PP2, GDC0941, or LY294002. (**E**) Migration rates measured in wound width (μm)/time (h) of non-irradiated mock-transduced A549 cells treated with PBS or COMP and either no additional treatment, cilengitide, PP2, GDC0941, or LY294002. (**F**) Migration rates of irradiated mock-transduced A549 cells treated with PBS or COMP and either no additional treatment, cilengitide, PP2, GDC0941, or LY294002. (**G**) Migration rates of non-irradiated mock-transduced NCI-H1437 cells treated with PBS or COMP and either no additional treatment, cilengitide, PP2, GDC0941, or LY294002. (**H**) Migration rates of irradiated mock-transduced NCI-H1437 cells treated with PBS or COMP and either no additional treatment, cilengitide, PP2, GDC0941, or LY294002. Statistical analysis done by one-way ANOVA followed by Tukey’s post-hoc test. Error bars represent mean ± SEM (* *p* < 0.05, ** *p* < 0.01, *** *p* < 0.001, **** *p* < 0.0001).

A marginal reduction in migration was observed after 2Gy in the Mock A549 cell line vs. non-irradiated Mock A549 cells (Figure 3B). Treatment with 2Gy marginally reduced migration in the COMP OE cells and vs. non-irradiated Mock A549 cells (Figure 3B). However, 2Gy did not reduce migration in Mock A549 cells that were treated with exogenous COMP (Figure 3B). Cilengitide treatment did not reduce migration rates in non-irradiated or irradiated COMP OE cells (Figure 3A,B). However, PP2 and GDC0941 treatment resulted in reduced migration after 2Gy in the COMP OE cells (*p* < 0.0001), as did LY294002 treatment (*p* < 0.01) (Figure 3B).

As a control, inhibitors were tested in the Mock A549 cell line. In these non-irradiated cells, proliferation and migration were not affected by COMP treatment alone, nor COMP treatment + Cilengitide (Figure 2E,F and Figure 3E,F). As noted, while 2Gy lowered proliferation and marginally reduced migration of Mock A549 cells vs. non-irradiated Mock A549 cells, this response was not observed with COMP treatment and 2Gy; proliferation and migration was not reduced. Cilengitide treatment did not alter this response (Figure 2F and Figure 3F). However, proliferation and migration rates were lowered in irradiated Mock A549 cells that were treated with both COMP and either PP2, GDC0941 or LY294002 (*p* < 0.0001) (Figure 2F and Figure 3F); effectively reversing the radioprotective effects of COMP.

### 2.4. COMP Overexpression in the NCI-H1437 Cell Line and the Response to Radiation with/Without COMP Signaling Inhibitors

In non-irradiated Mock transduced NCI-H1437 cells, treatment with exogenous COMP reduced proliferation rates vs. Mock NCI-H1437 control (*p* < 0.0001) (Figure 2C). However, overexpression of COMP (COMP OE) did not reduce proliferation (Figure 2C). COMP OE NCI-H1437 cells treated with 2Gy did not have reduced proliferation (Figure 2D). Cilengitide treatment reduced proliferation in the COMP OE NCI-H1437 cell line after 2Gy (*p* < 0.001); as did GDC0941 treatment (*p* < 0.05) (Figure 2D). Treatment with PP2 or LY294002 only slightly decreased proliferation after 2Gy in COMP OE NCI-H1437 cells (Figure 2D).

Exogenous COMP treatment in non-irradiated Mock NCI-H1437 cells marginally reduced migration vs. Mock control NCI-H1437 cells (Figure 3G). However, COMP OE NCI-H1437 cells did not experience this reduction (Figure 3C). Mock NCI-H1437 cells treated with 2Gy experienced a slight decrease in migration (Figure 3D). COMP treatment did not affect the 2Gy-induced reduction in migration in Mock NCI-H1437 cells (Figure 3D). COMP OE NCI-H1437 also experienced a slight decrease in migration vs. non-irradiated Mock NCI-H1437 cells (Figure 3D). PP2 treatment marginally decreased migration in irradiated COMP OE NCI-H1437 cells (Figure 3D). However, a reduction in migration was not seen in irradiated COMP OE NCI-H1437 cells treated with cilengitide, GDC0941, or LY294002 (Figure 3D).

As a control, the inhibitors were tested in the NCI-H1437 mock-transduced cell line as well. In these non-irradiated cells, reduced proliferation was exhibited after COMP treatment alone, COMP treatment + cilengitide, COMP treatment + GDC0941, or COMP treatment + LY294002 (Figure 2G). Marginally reduced migration rates were exhibited in Mock NCI-H1437 cells treated with both COMP and cilengitide, GDC0941, or LY294002 vs. COMP treatment alone (Figure 3G). As noted, COMP treatment and 2Gy slightly reduced proliferation of Mock NCI-H1437 cells, and further reduction in proliferation was exhibited in irradiated Mock NCI-H1437 cells treated with both COMP and either cilengitide, PP2, or GDC0941 (Figure 2H). As stated, COMP treatment did not affect the 2Gy-induced reduction in migration, and treatment with both COMP and any of the inhibitors also did not affect this reduction in migration (Figure 3H).

### 2.5. COMP Overexpression and the Invasion Response to Radiation with or Without COMP Signaling Inhibitors

**Figure 4:**
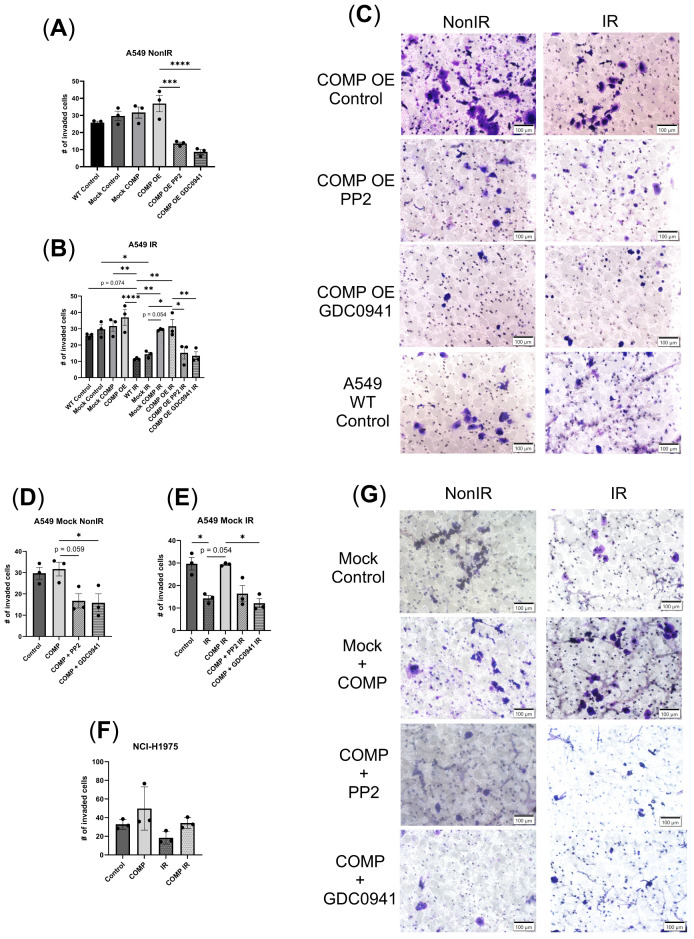
Exogenous COMP treatment and COMP overexpression mitigate radiation-induced reduction of invasion. (**A**) The number of invaded cells of non-irradiated A549 wild-type (WT), mock-transduced (Mock), and COMP-overexpressed (COMP OE) cells that were treated with either PBS (control), COMP, COMP and PP2 (PP2) or COMP and GDC0941 (GDC0941). (**B**) The number of invaded cells of irradiated A549 wild-type, mock-transduced, and COMP-overexpressed cells that were treated with either PBS (control), COMP, COMP and PP2 (PP2), or COMP and GDC0941 (GDC0941) compared with the number of invaded cells of non-irradiated cells from (**A**). (**C**) Representative images of invaded cells of COMP-overexpressed (COMP OE) A549 and A549 wild-type cells that were irradiated or non-irradiated and either treated with PBS (control), PP2 or GDC0941. (**D**) The number of invaded cells of non-irradiated mock-transduced (Mock) A549 cells treated with either PBS (control), COMP, COMP and PP2 (PP2), or COMP and GDC0941 (GDC0941). (**E**) The number of invaded cells of irradiated mock-transduced A549 cells treated with either PBS (control), COMP, COMP and PP2 (PP2), or COMP and GDC0941 (GDC0941). (**F**) The number of invaded cells of non-irradiated and irradiated NCI-H1975 cells treated with either PBS (control) or COMP. (**G**) Representative images of invaded cells of mock-transduced A549 cells that were irradiated or non-irradiated and treated with either PBS (control), COMP, COMP and PP2, or COMP and GDC0941.Images taken at 20×. Statistical analysis done by one-way ANOVA followed by Tukey’s post-hoc test. Individual points on graphs represent average number of invaded cells counted in four 20× images. N = 3. Error bars represent mean ± SD (* *p* < 0.05, ** *p* < 0.01, *** *p* < 0.001, **** *p* < 0.0001).

Invasiveness of COMP OE A549 cells exhibited marginally increased numbers of invaded cells (+43.4% A549 COMP OE vs. A549 wild-type (WT) Control) (Figure 4A,C). PP2 treatment reduced invasion in COMP OE A549 cells (*p* < 0.001); as did GDC0941 treatment (*p* < 0.0001) (Figure 4A,C). A549 WT cells had reduced invasion after 2Gy vs. non-irradiated A549 WT cells (*p* = 0.074); this 2Gy-induced decrease in invasion also occurred in Mock A549 cells (*p* < 0.05) (Figure 4B,C). However, COMP OE did not exhibit decreased invasion after 2Gy vs. non-irradiated COMP OE cells (Figure 4B,C). Irradiated mock A549 cells with exogenous COMP treatment also did not exhibit decreased invasion after 2Gy vs. non-irradiated mock A549 cells (Figure 4B). PP2 treatment reduced invasion in COMP OE A549 cells after 2Gy (*p* < 0.05); as did GDC0941 treatment (*p* < 0.01) (Figure 4B,C).

As a control, Mock A549 cells were treated with the COMP signaling inhibitors. In the non-irradiated cells, PP2 + COMP treatment reduced invasion vs. COMP treatment alone (*p* = 0.059); as did GDC0491 + COMP treatment (*p* < 0.05) (Figure 4D,G). GDC0941 treatment with exogenous COMP decreased invasion vs. COMP treatment alone after 2Gy (*p* < 0.05) (Figure 4E,G); and PP2 + COMP treatment after 2Gy had a similar reduction (Figure 4E,G).

### 2.6. Exogenous COMP Treatment or COMP Overexpression Reduce Radiation-Induced Cell Death

**Figure 5:**
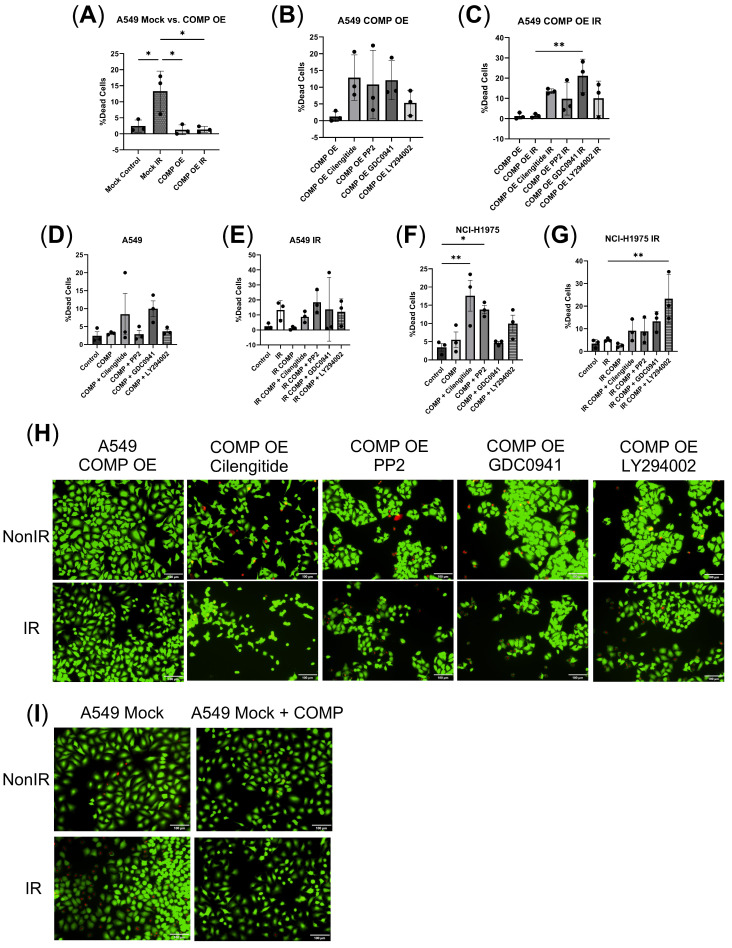
Exogenous COMP treatment and COMP overexpression prevent loss of viability due to radiation. (**A**) The percentage of dead mock-transduced (Mock) and COMP-overexpressed (COMP OE) when irradiated and non-irradiated. (**B**) The percentage of dead COMP-overexpressed (COMP OE) A549 cells 24 h after treatment with either PBS (control), cilengitide, PP2, GDC0941, or LY294002. (**C**) The percentage of dead COMP-overexpressed irradiated A549 cells after treatment with either PBS (control), cilengitide, PP2, GDC0941, or LY294002. (**D**) The percentage of dead mock-transduced A549 cells 24 h after treatment with either PBS (control), COMP, cilengitide, PP2, GDC0941, or LY294002. (**E**) The percentage of dead irradiated mock-transduced A549 cells 24 h after treatment with either PBS (control), COMP, cilengitide, PP2, GDC0941, or LY294002. (**F**) The percentage of dead NCI-H1975 cells 24 h after treatment with either PBS (control), COMP, cilengitide, PP2, GDC0941, or LY294002. (**G**) The percentage of dead irradiated NCI-H1975 cells 24 h after treatment with either PBS (control), COMP, cilengitide, PP2, GDC0941, or LY294002. (**H**) Representative images of live (green) and dead (red) A549 COMP-overexpressed cells when irradiated or non-irradiated and after treatment with either PBS (control), cilengitide, PP2, GDC0941, or LY294002. (**I**) Representative images of live (green) and dead (red) mock-transduced A549 cells when irradiated or non-irradiated and after treatment with either PBS (control) or COMP. Images taken at 20×. Statistical analysis done by one-way ANOVA followed by Tukey’s or Dunnett’s multiple comparisons test. Individual points on graph represent the average % of dead cells counted per treatment which was conducted in duplicate. N = 3. Error bars represent mean ± SD (* *p* < 0.05, ** *p* < 0.01).

In A549 COMP overexpressed cells, cilengitide, PP2 or GDC0941 treatment marginally increased the percentage of dead cells vs. control (Figure 5B,H). Irradiated mock-transduced A549 cells exhibited a higher percentage of dead cells vs. nonirradiated mock cells (*p* < 0.05) (Figure 5A,H). Irradiated COMP overexpressed cells exhibited no change in the percentage of dead cells vs. nonirradiated mock cells, however cilengitide, PP2, and LY294002 treatment marginally elevated and GDC0941 treatment increased the percentage of dead cells (*p* < 0.01) (Figure 5C,H).

As a control, the inhibitors were tested in the mock transduced A549 and NCI-H1975 cell lines. In non-irradiated mock transduced A549 cells, cilengitide + COMP and GDC0941 + COMP treatment marginally increased the percentage of dead cells (Figure 5D). While in non-irradiated NCI-H1975 cells, cilengitide + COMP (*p* < 0.01) and PP2 + COMP (*p* < 0.05) treatment increased the percentage of dead cells (Figure 5F); and LY294002 + COMP treatment marginally elevated the percentage of dead cells (Figure 5F). As noted, irradiated mock-transduced A549 cells had an elevated percentage of dead cells vs. non-irradiated cells; however, this was not exhibited in irradiated COMP treated A549 cells or NCI-H1975 cells (Figure 5E,F,I). In irradiated mock transduced A549 cells, cilengitide + COMP, PP2 + COMP, GDC0941 + COMP, and LY294002 + COMP treatment marginally increased the percentage of dead cells vs. COMP treatment alone (Figure 5E). LY294002 + COMP treatment increased the percentage of dead cells in irradiated NCI-H1975 cells vs. irradiated COMP treated NCI-H1975 cells (*p* < 0.01) (Figure 5G); and cilengitide + COMP, PP2 + COMP, and GDC0941 + COMP marginally elevated the percentage of dead cells (Figure 5G).

### 2.7. Comparative Proteomic Analysis of Non-Irradiated and Irradiated A549 Cells Treated with COMP and COMP Inhibitors

**Figure 6:**
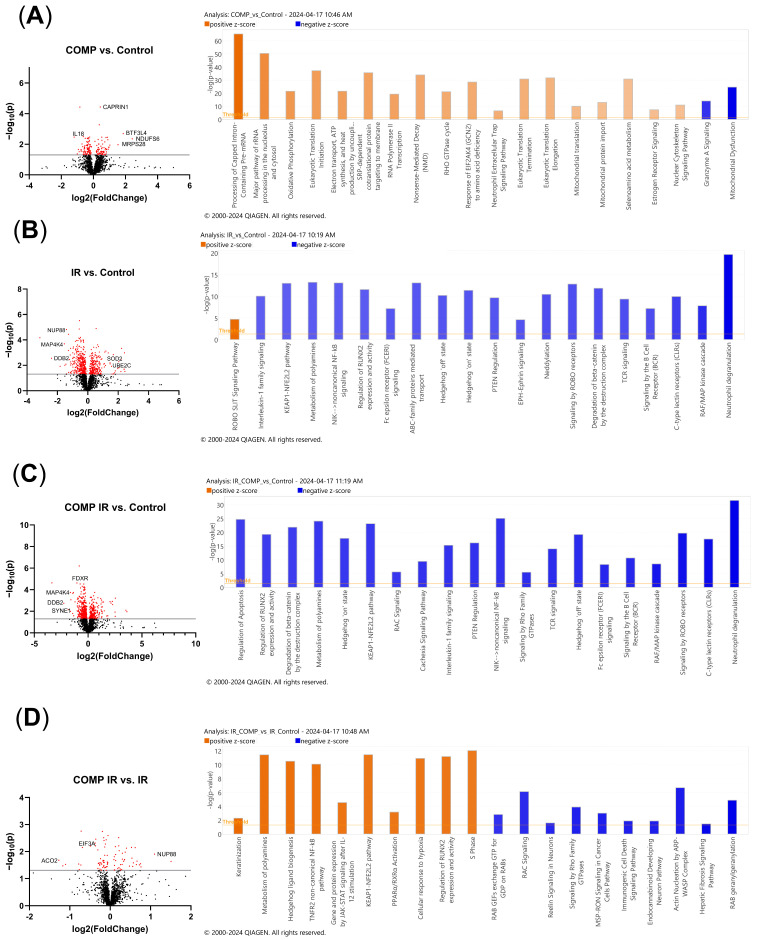

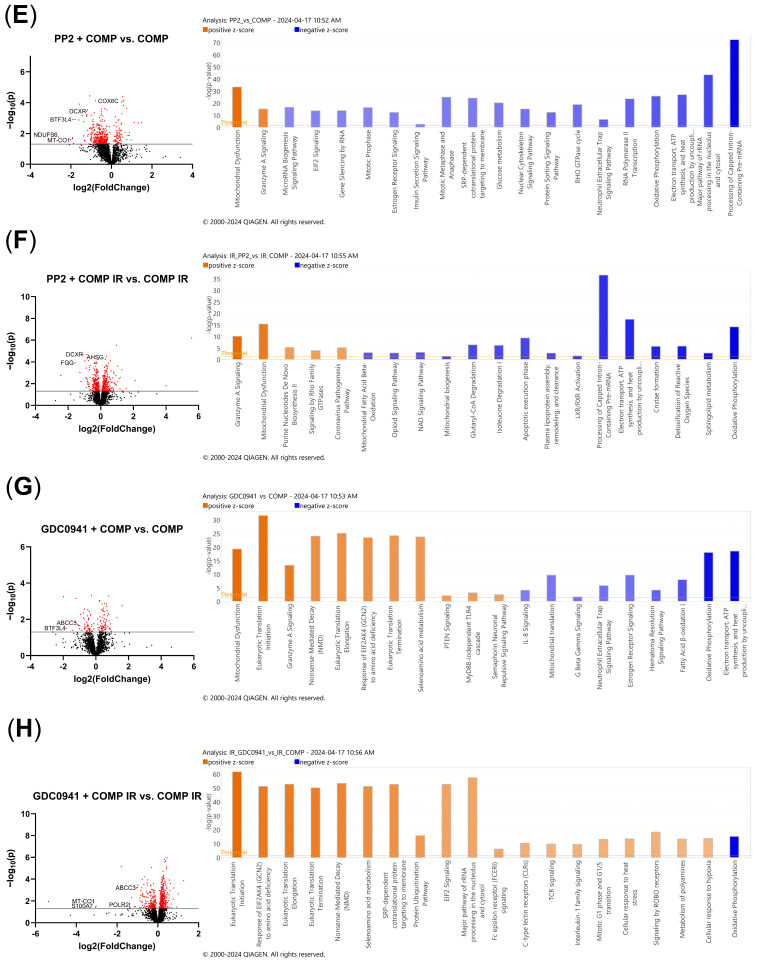
Pathway analysis and protein expression indicate a pro-oxidative phosphorylation and anti-mitochondrial dysfunction phenotype in COMP-treated NSCLC cells. (**A**) Comparative analysis of COMP-treated vs. Control A549 cells identified 160 significantly altered proteins (*p* < 0.05). Most significantly altered pathways from comparative analysis of COMP-treated vs. Control A549 cells (Z-score > ±2, *p* < 0.001). (**B**) Comparative analysis of IR vs. Control A549 cells identified 617 significantly altered proteins (*p* < 0.05). Most significantly altered pathways from comparative analysis of IR vs. Control A549 cells (Z-score > −2, *p* < 0.001). (**C**) Comparative analysis of IR COMP-treated vs. Control A549 cells identified 587 significantly altered proteins (*p* < 0.05). Most significantly altered pathways from comparative analysis of IR COMP-treated vs. Control A549 cells (Z-score > ±2, *p* < 0.001). (**D**) Comparative analysis of IR COMP-treated vs. IR A549 cells identified 89 significantly altered proteins (*p* < 0.05). Most significantly altered pathways from comparative analysis of IR COMP-treated vs. IR A549 cells (Z-score > ±2, *p* < 0.001). (**E**) Comparative analysis of PP2 + COMP treated vs. COMP-treated A549 cells identified 448 significantly altered proteins (*p* < 0.05). Most significantly altered pathways from comparative analysis of PP2 + COMP treated vs. COMP-treated A549 cells (Z-score > ±2, *p* < 0.001). (**F**) Comparative analysis of IR PP2 + COMP treated vs. IR COMP-treated A549 cells identified 481 significantly altered proteins (*p* < 0.05). Most significantly altered pathways from comparative analysis of PP2 + COMP treated vs. IR COMP-treated A549 cells (Z-score > ±2, *p* < 0.001). (**G**) Comparative analysis of GDC0941 + COMP treated vs. COMP-treated A549 cells identified 136 significantly altered proteins (*p* < 0.05). Most significantly altered pathways from comparative analysis of GDC0941 + COMP treated vs. COMP-treated A549 cells (Z-score > ±2, *p* < 0.001). (**H**) Comparative analysis of IR GDC0941 + COMP treated vs. IR COMP-treated A549 cells identified 527 significantly altered proteins (*p* < 0.05). Most significantly altered pathways from comparative analysis of IR GDC0941 + COMP treated vs. IR COMP-treated A549 cells (Z-score > ±2, *p* < 0.001). The red color indicates that the False Discovery Rate (FDR) is >2 and the black color indicates that the FDR < 2. The grey indicates FDR = 2. FDR scores of 2 or greater are considered significant.

Comparative analysis of protein expression in COMP-treated A549 cells vs. control cells identified 160 significantly altered proteins (*p* < 0.05) (Figure 6A). Pro-tumorigenic enriched differential protein expression of BTF3L4, MRPS28, and CAPRIN1, decreased expression of IL18, and pro-oxidative phosphorylation increased expression of NDUFS6 was observed (Figure 6A). Irradiated A549 cells vs. control identified 617 significantly altered proteins (*p* < 0.05) (Figure 6B). Pro-tumorigenic enriched differential protein expression of UBE2C, anti-tumorigenic enriched differential protein expression of MAP4K4 and NUP88, pro-DNA damage response enriched protein expression of SOD2 and decreased expression of DDB2 was observed (Figure 6B). Irradiated COMP-treated A549 cells vs. non-irradiated control identified 587 significantly altered proteins (*p* < 0.05) (Figure 6C). Decreased expression of pro-DNA damage response protein DDB2, decreased expression of pro-tumorigenic proteins MAP4K4 and SYNE1, and decreased expression of pro-apoptotic protein FDXR was observed (Figure 6C). Irradiated COMP-treated A549 cells vs. irradiated cells identified 89 significantly altered proteins (*p* < 0.05) (Figure 6D). Pro-tumorigenic enriched differential protein expression of NUP88, decreased expression of anti-tumorigenic protein ACO2 and decreased expression of translation factor of DNA repair proteins, EIF3A was observed (Figure 6D).

Comparative analysis of protein expression in PP2 + COMP treated vs. COMP treated A549 cells identified 448 significantly altered proteins (*p* < 0.05) (Figure 6E). Decreased expression of pro-tumorigenic proteins BTF3L4, Cox6C, and DCXR, and decreased expression of pro-oxidative phosphorylation proteins NDUFS6 and MT-CO1 was observed (Figure 6E). Irradiated PP2 + COMP treated vs. irradiated COMP treated A549 cells identified 481 significantly altered proteins (*p* < 0.05) (Figure 6F). Decreased expression of pro-tumorigenic proteins DCXR, AHSG and FGG was observed (Figure 6F). Non-irradiated GDC0941 + COMP treated vs. COMP treated A549 cells identified 136 significantly altered proteins (*p* < 0.05) (Figure 6G). Decreased expression of pro-tumorigenic proteins ABCC3 and BTF3L4 was observed (Figure 6G). Irradiated GDC0941 + COMP treated vs. irradiated COMP treated A549 cells identified 527 significantly altered proteins (*p* < 0.05) (Figure 6H). Decreased expression of pro-tumorigenic proteins ABCC3 and S100A7, decreased expression of pro-treatment resistance protein POLR2I, and decreased expression of oxidative phosphorylation protein MT-CO1 was observed (Figure 6H).

### 2.8. Proteomic Pathway Analysis of Non-Irradiated and Irradiated A549 Cells Treated with COMP and COMP Inhibitors to Characterize Intracellular Responses

Comparative proteomic pathway analysis of COMP treated A549 cells vs. control A549 cells without exogenous COMP indicated upregulated pathways in translation initiation, elongation, processing of capped intron-containing pre-mRNA, oxidative phosphorylation, EIF2 signaling, and mitochondrial translation and protein import and decreased markers associated with mitochondrial dysfunction, and granzyme A signaling (Z-score > ±2, *p* < 0.001) (Figure 6A). Irradiated A549 cells vs. nonirradiated cells, indicated a downregulation of the KEAP1-NFE2L2 pathway, PTEN regulation, IL-1 family signaling, RAF/MAP kinase cascade, regulation of RUNX2 expression and activity, non-canonical NF-kB signaling, and the hedgehog ‘on’ state (Z-score > ±2, *p* < 0.001) (Figure 6B). Irradiated COMP treated A549 cells vs. non-irradiated control cells, indicated a downregulation of regulation of apoptosis, regulation of RUNX2 expression and activity, hedgehog ‘on’ state, KEAP1-NFE2L2 pathway, Interleukin-1 family signaling, PTEN regulation, non-canonical NF-kB signaling, and the RAF/MAP kinase cascade (Z-score > ±2, *p* < 0.001) (Figure 6C). Irradiated COMP treated A549 cells vs. irradiated A549 cells indicated an upregulation of pathways including metabolism of polyamines, hedgehog ligand biogenesis, non-canonical NF-kB signaling, gene and protein expression by JAK-STAT signaling, KEAP1-NFE2L2 pathway, regulation of RUNX2 expression and activity and the S phase of mitosis; and a downregulation of the immunogenic cell death signaling pathway (Z-score > ±2, *p* < 0.001) (Figure 6D).

Similar to protein expression, comparative pathway analysis of COMP signaling inhibitors in COMP-treated A549 cells was performed. Nonirradiated PP2 + COMP treated A549 cells vs. COMP treatment alone indicated an upregulation of mitochondrial dysfunction and granzyme A signaling and a downregulation of pathways including EIF2 signaling, estrogen receptor signaling, glucose metabolism, mitotic metaphase and anaphase, oxidative phosphorylation, electron transport and ATP synthesis, and processing of rRNA and pre-mRNA (Z-score > ±2, *p* < 0.001) (Figure 6E). Irradiated PP2 + COMP treated A549 cells vs. irradiated COMP treated cells indicated an upregulation of granzyme A signaling and mitochondrial dysfunction and a downregulation of mitochondrial fatty acid beta-oxidation, NAD signaling pathway, mitochondrial biogenesis, processing of capped intron pre-mRNA, electron transport and ATP synthesis, detoxification of reactive oxygen species, and oxidative phosphorylation (Z-score > ±2, *p* < 0.001) (Figure 6F). Non-irradiated GDC0941 + COMP treated A549 cells vs. COMP treatment alone indicated an upregulation of pathways mitochondrial dysfunction, granzyme A signaling, nonsense-mediated decay and PTEN signaling, and a downregulation of mitochondrial translation, estrogen receptor signaling, fatty acid beta-oxidation, oxidative phosphorylation and electron transport and ATP synthesis (Z-score > ±2, *p* < 0.001) (Figure 6G). Irradiated GDC0941 + COMP treated A549 cells vs. irradiated COMP treated cells indicated an upregulation of pathways nonsense-mediated decay, protein ubiquitination pathway, IL-1 signaling, and a decrease in oxidative phosphorylation (Z-score > ±2, *p* < 0.001) (Figure 6H).

## 3. Discussion

Identifying new treatment strategies for patients with NSCLC is crucial, as lung cancer is the leading cause of cancer-related deaths in the US [1]. Specifically, identifying novel biomarkers of NSCLC progression and treatment resistance is important for informing doctors and patients when deciding upon treatment plans. In our study, we observed that treatment of NSCLC cells with exogenous COMP or overexpression of COMP in A549 cells affected the sensitivity of these cells to radiation. Importantly, this study identified that: (1) treatment of NSCLC cells with exogenous COMP prior to irradiation increased the proliferation, migration and invasion ability of cells compared to irradiated cells alone; (2) overexpression of COMP in NSCLC cells increased the proliferation, migration and invasion ability when irradiated compared to irradiated mock-transduced cells; and (3) the radioprotective effect imparted by both COMP overexpression and exogenous COMP treatment was reduced or prevented with inhibitors of Src or PI3k (downstream intermediates of COMP signaling). Additionally, the addition of exogenous COMP to NSCLC cells did not significantly increase the proliferation, migration, invasion or viability of wild-type or mock-transduced NonIR NSCLC cells. Proliferation and invasion were marginally greater in the non-irradiated cells with COMP overexpression, similar to previous studies that identified COMP expression in breast and prostate cancer promotes invasion and growth [12,17]. The COMP treatment and overexpression seem to have greatest effect on tumor responses when irradiated, and as such the degree of COMP expression in NSCLC may serve as a biomarker that predicts response to radiation therapy.

COMP-mediated radiation resistance was observed for the A549 and NCI-H1975 cells, but not the NCI-H1437 cell line. Exogenous COMP treated mock-transduced NCI-H1437 cells exhibited reduced proliferation and migration rates regardless of whether cells were irradiated or not; overexpressing COMP did not reduce proliferation and migration and instead maintained rates. Unlike the other cell lines, the NCI-H1437 cell line does not express COMP, whereas the other cell lines do express COMP [24]. Importantly, COMP is upregulated in NSCLC adenocarcinoma tumor tissue compared to “normal” lung tissue (*p* < 0.05) (Figure S2). Thus, unlike other cell lines and NSCLC in patients that do express COMP, the response of NCI-H1437 to exogenous COMP signaling may not be translatable. The responses of NCI-H1437 to COMP indicate that COMP negative tumors may not be radioresistant, further enhancing our findings that COMP-overexpression in NSCLC promotes radiation resistance.

The proteome of COMP-treated cells, both irradiated and non-irradiated, identify increased concentration of proteins associated with elevated tumor proliferation and aggressiveness. Non-irradiated COMP-treated A549 cells exhibited higher protein expression of BTF3L4, a protein implicated in non-small cell lung cancer proliferation and progression [25,26], and NDUFS6, a mitochondrial protein associated with elevated tumorigenesis and decreased mitochondrial apoptosis [27,28]; these proteins had lower expression in PP2 + COMP treated and GDC0941 + COMP treated cells. Pathway analysis indicated non-irradiated COMP treated cells exhibited upregulated oxidative phosphorylation and downregulated mitochondrial dysfunction; while PP2 + COMP treated and GDC0941 + COMP treated cells exhibited the opposite. Additionally, Nup88, a nuclear pore protein associated with migration, invasion and tumorigenesis [29,30], had decreased expression in irradiated A549 cells versus nonirradiated cells, but was upregulated in COMP-treated irradiated cells versus irradiated cells without exogenous COMP treatment. Pathway analysis of irradiated COMP treated A549 cells indicated upregulated hedgehog ligand biogenesis pathways, which is involved with lung cancer progression and drug resistance [31], and Keap1-NFE2L2 pathways which is associated with radiation resistance of lung cancer [32]. Irradiated PP2 and COMP-treated A549 cells exhibited lower protein concentration of DCXR, a protein associated with increased proliferation and progression of various cancer types [33,34]. Irradiated GDC0941 and COMP-treated GDC0941 and COMP-treated cells exhibited lower protein expression of ABCC3, a protein involved in treatment resistance in NSCLC and other cancers [35,36,37], and MT-CO1, a mitochondrial protein associated with cancer progression [38]. Therefore, mitochondrial function and oxidative phosphorylation may play a role in the increased progression of COMP treated cells and radiation resistance, which has previously been implicated in cancer drug and radio-resistance [39,40].

Our findings suggest that elevated levels of COMP expression may be used as a biomarker for patients with radiation resistant NSCLC, and downstream signaling intermediates of COMP could be targeted to reverse the radiation resistance conferred by COMP. Our study found that targeting Src and PI3K may rescue COMP-induced radiation sensitivity in NSCLC cells and could be a potential avenue for clinicians when determining treatment plans for patients. Src has been found to be involved in activating non-homologous end joining through the 53BP1 protein, which leads to increased DNA-damage repair [27]. Studies have also identified PI3k/AKT pathways inhibit apoptosis, through inhibition of caspase-9 and Bad [41], and activates DNA-damage repair through regulation of proteins such as p53, BRCA1 and HMGB1 [42]. Inhibitors against Src [43,44,45,46] and PI3K [47,48,49] have already been in clinical trials for patients with solid tumors and other cancer types such as breast cancer including GDC0941 [50,51], which was used in our study. A clinical trial to treat NSCLC patients that have a high expression of COMP with these inhibitors, prior to the start of radiation treatment, could be a future direction in order to assess whether these inhibitors would work clinically to reduce radiation resistance in NSCLC.

However, this study is not without limitations. In vitro assays such as the proliferation, migration, invasion and viability studies performed are not the best models for disease seen in the clinic, and may be subject to any numerous technical issues as with any in vitro experiments. Additionally, we only performed proteomics studies on cells treated with exogenous COMP, further omics studies on COMP-overexpressed cells and tumors should be considered, as could directly measuring DNA damage/protection. Future studies should also look at the effects seen in our in vitro studies and see if the same observations can be made in an in vivo study, and with retrospective or prospective clinical studies in patients. With these limitations, we still believe that our findings are useful in informing future studies and insights into radiation resistant NSCLC.

Our study importantly demonstrates that COMP could serve as a biomarker of radiation resistance and progression in NSCLC. We also demonstrate that this resistance could be reversed by targeting downstream intermediates of COMP signaling. We aim to further examine COMP in the context of radiation resistance NSCLC through clinical studies.

## 4. Materials and Methods

### 4.1. TCGA Data

COMP expression from 483 lung adenocarcinoma tumors and 347 “normal” lung tissues was obtained from The Cancer Genome Atlas (TCGA) database using the GEPIA2 online tool [52]. COMP expression in tumor tissue was compared to “normal” tissue to demonstrate how COMP may be an important protein to consider in lung cancer. The GEPIA2 tool utilizes RNA-seq datasets of samples from the UCSC Xena project and data normalized using Quantile-normalization. COMP expression was analyzed by ANOVA using a Log_2_FC cutoff of 1 and *p*-value cutoff of 0.1.

### 4.2. Cell Culture, Transduction, and RT-qPCR Verification

A549, NCI-H1975, and NCI-H1437 cell lines were purchased from the American Type Culture Collection (ATCC). All experiments were performed on cultures from these aliquots within no more than 10 passages from purchase. All cell lines were grown in RPMI 1640 medium (Gibco, Waltham, MA, USA) supplemented with 10% FBS, penicillin (100 U/mL) and streptomycin (100 µg/mL), and L-glutamine, in a humidified incubator at 5% CO_2_ at 37 °C. Media was obtained from the Wake Forest Cell Engineering Shared Resource.

Full-length COMP or mock (empty vector) were stably expressed in A549 and NCI-H1437 cell lines by lentiviral transduction (Origene, Rockville, MD, USA) and selection with puromycin (Gibco). The NCI-H1437 cell line was used as a negative control for COMP expression as it does not express COMP. COMP expression was verified by real-time quantitative PCR (RT-qPCR). Cells were incubated in 6 well plates for 2 days, followed by cell lysate collection, RNA extraction (RNeasy Plus Mini Kit, Qiagen, Rockville, MD, USA), cDNA synthesis (High Capacity RNA to cDNA kit, ThermoFisher, Waltham, MA, USA), and RT-qPCR (QuantStudio3, Applied Biosystems, Waltham, MA, USA) using Sybr Green PCR Master Mix (AppliedBiosystems, Waltham, MA, USA). Primers used for RT-qPCR were COMP (fwd: GACAGTGATGGCGATGGTATAG, rev: TCACAAGCATCTCCCACAAA), GAPDH (fwd: GGTGTGAACCATGAGAAGTATGA, rev: GAGTCCTTCCACGATACCAAAG) and TBP (fwd: CCACTCACAGACTCTCACAAC, rev: TGCGGTACAATCCCAGAAC) (IDT, Coralville, IA, USA). GADPH and TBP were used as reference genes. Fold change was calculated using the 2^−∆∆Ct^ method [53]. All RT-qPCR reactions were conducted in triplicate.

### 4.3. Indirect COMP Inhibitor Treatments

PP2 was purchased from Sigma-Aldrich (St. Louis, MO, USA) (#529573). All other inhibitors were purchased from Selleckchem (Houston, TX, USA). GDC-0941 (#S1065) is considered a pan-PI3K inhibitor with high selectivity against PI3Kα/δ and modest selectivity against p110β and p110γ. Cilengitide (#S7077) is a nonselective inhibitor of αVβ3 and αVβ5 integrins. PP2 is a potent, reversible, ATP-competitive, inhibitor of the Src family of protein tyrosine kinases. LY294002 (#S1105) is a strong, non-selective inhibitor of PI3Ks. All concentrations of inhibitors used were based on preliminary proliferation results to determine the approximate concentration needed to reduce proliferation to 50% of the baseline rate. Cells were exposed to varying concentrations of: cilengitide (0 µM, 0.25 µM, 0.5 µM, 0.75 µM, 1 µM, or 5 µM), PP2 (0 µM, 0.1 µM, 0.25 µM, 0.5 µM, 1 µM, or 2 µM), GDC0941 (0 µM, 1 µM, 5 µM, or 10 µM), or LY294002 (0 µM, 1 µM, 5 µM, 10 µM, 20 µM).

### 4.4. Proliferation Assay

A549, NCI-H1975 and NCI-H1437 cells were seeded at a density of 4000 cells/well in clear 96-well plates (Corning, Lowell, MA, USA). HCC826 cells were seeded at a density of 2000 cells/well. After cells adhered to the plate, about 4–6 h later, treatments were added to plates in duplicate. Treatments included: rhCOMP (2 µg/mL), cilengitide (1 µM), PP2 (1 µM), GDC0941 (330 nM), and LY294002 (2.5 µM) with PBS used as a control. Inhibitor treatments were given alone and in combination with COMP. 12 h after treatment, cells were either irradiated with 2Gy using a Cs137 irradiator or not irradiated. 2Gy was chosen as it is a standard fractionation dose given to patients with locally advanced NSCLC and has previously been shown to reduce the proliferation of lung cancer in vitro [54,55]. After irradiation, plates were placed in the IncuCyte ZOOM live cell imager equipped with a Nikon Camera (10× objective) for 48 h. The IncuCyte ZOOM Software v2016A (Essen Bioscience, Ann Arbor, MI, USA) was used to quantify and capture proliferation every 2 h. Proliferation experiments were performed three times.

### 4.5. Migration Assay

A549, NCI-H1975 and NCI-H1437 cells were seeded at a density of 40,000 cells/well in Incucyte^®^ Imagelock 96-Well Plates (Sartorius, Bohemia, NY, USA). After cells adhered to the plate, about 4–6 h later treatments were added to the plates in duplicate the same as in the proliferation assay described above. 12 h after treatment, cells were either irradiated with 2Gy using a Cs137 irradiator or not irradiated. The Incucyte^®^ 96-Well Woundmaker Tool was then used to create a scratch wound. After scratching, the wells were washed with PBS, and treatments and media were readded to the wells. Then, the plates were placed into the IncuCyte ZOOM live cell imager for 48 h. The IncuCyte ZOOM Software (Essen Bioscience, Ann Arbor, MI, USA) was used to quantify and capture migration every 2 h. Scratch wound width in microns was used to evaluate migration. Migration experiments were performed three times.

### 4.6. Invasion Assay

A549 and NCI-H1975 cells were plated at a density of 5 × 10^4^ cells/well in 48 well plates. Cells were pre-incubated for 1 h as done in Englund et al. 2017 [12] in at 37 °C 5% CO_2_ with either PBS or COMP (2 µg/mL). Cells with COMP either were treated with no additional treatment, PP2 (1 µM), or GDC0941 (330 nM). After 1 h, cells were either irradiated with 2Gy using a Cs137 irradiator or not irradiated. Cells were then transferred to inserts of the Corning BioCoat Matrigel Invasion Chamber, 8.0 µm pore size in singlet. Full media with 10% FBS was put in the bottom of the transwell plate to act as an attractant. Cells were then incubated and allowed to invade for 24 h. After 24 h, wells were carefully washed with PBS and cells were removed from the apical side of the transwell with a cotton tip [56]. Invaded cells were then fixed with ice-cold methanol, stained with 0.25% crystal violet and allowed to dry overnight [12]. Four random images were taken of each well at 20× using an Olympus IX70 inverted microscope (Olympus Corporation, Center Valley, PA, USA) and cells were counted in each image and averaged using ImageJ v1.52q. This assay was performed three times.

### 4.7. Live/Dead Assay

A549, NCI-H1975 and COMP+ A549 cells were plated at a density of 1.5 × 10^4^ cells/well in 96 well plates and treated with the same treatments as in the proliferation assay in quadruplicate. 4 h after treatment, half of the cells were irradiated with 2Gy using a linear accelerator (Elekta, Stockholm, Sweden) while the other half were not irradiated. 24 h after treatment, the live/dead assay was performed using the Invitrogen LIVE/DEAD™ Viability/Cytotoxicity Kit (Invitrogen, Carlsbad, CA, USA), for mammalian cells and 3 fluorescent images per group were taken at 20× using an Olympus IX70 inverted fluorescence microscope (Olympus Corporation, Center Valley, PA, USA). Total number of live and dead cells per image were counted using ImageJ v1.52q, and averaged to calculate the percent of live cells and percent of dead cells. This assay was performed three times.

### 4.8. Proteomics

A549 cells were plated at a density of 3 × 10^5^ cells/well in Corning 6-well plates. Cells were allowed to adhere and then treated with either COMP (2 µg/mL), or PBS as a control. PP2 (1 µM) or GDC0941 (330 nM) was added to wells in addition to COMP. 12 h after treatment, cells were either irradiated with 2Gy using a Cs137 irradiator or not irradiated. Cells were then incubated for 24 h. After 24 h, cells were trypsinized, spun down into a cell pellet, and frozen at −80 °C for mass spectrometry analysis. Four replicates of each treatment were collected for analysis. Samples were analyzed on a LC-MS/MS system consisted of an Orbitrap Eclipse Mass Spectrometer (Thermo Scientific, Waltham, MA, USA) and a Vanquish Neo nano-UPLC system (Thermo Scientific, Waltham, MA, USA). Peptides were separated on a DNV PepMap Neo (1500 bar, 75 μm × 500 mm) column (Thermo Scientific, Waltham, MA, USA) for 120 min employing linear gradient elution consisted of water (A) and 80% acetonitrile (B) both of which contained 0.1% formic acid. Data were acquired by top speed data dependent mode where maximum MS/MS scans were acquired per cycle time of 1 s between adjacent survey spectra. Dynamic exclusion option was enabled which duration was set to 120 s. To identify proteins, spectra were searched against the UniProt human protein FASTA database (20,395 annotated entries, June 2021) using the Sequest HT search engine with the Proteome Discoverer v2.5 (Thermo Scientific, Waltham, MA, USA). Search parameters were as follows: FT-trap instrument; parent mass error tolerance, 10 ppm; fragment mass error tolerance, 0.6 Da (monoisotopic); enzyme, trypsin (full); # maximum missed cleavages, 2; variable modification, +15.995 Da (oxidation) on methionine; static modification, +57.021 Da (carbamidomethyl) on cysteine.

### 4.9. Statistical Analysis

An unpaired *t*-test was used to evaluate the RT-qPCR data as well as the TCGA data. Linear regressions were used to evaluate proliferation and migration curves and to calculate slopes of the curves. The differences in the slopes of the regressions and differences in invasion were tested between groups using a one-way ANOVA followed by Tukey post-hoc test for multiple comparisons or an unpaired *t*-test. Differences in percentage of dead cells in the viability assay was tested between groups using a one-way ANOVA followed by Dunnett’s post-hoc test. All tests except for proteomic analysis were performed with GraphPad Prism v10.2.0 (San Diego, CA, USA). For proteomic analyses, differentially expressed protein that were statistically significant defined as *p* ≤ 0.05 after adjusting for false discovery (FDR) were analyzed using Ingenuity Pathway Analysis (IPA).

## 5. Conclusions

In conclusion, our study suggests COMP is involved in NSCLC radiation resistance. Our study also provides a potential therapeutic treatment strategy for NSCLC patients with high expression of COMP through the use of Src or PI3k inhibitors currently in clinical use or clinical trials. Further research is required to fully elucidate the effects of COMP in NSCLC and explore the role of COMP in a clinical setting.

## Data Availability

The raw data supporting the conclusions of this article are housed within the private network of the Wake Forest University School of Medicine system and can be made available by the authors upon request. Data in Figure S2 are based upon openly available data generated by the TCGA Research Network: https://www.cancer.gov/tcga (accessed on 23 August 2023).

## Supplementary Materials

The following supporting documents can be downloaded at https://www.mdpi.com/article/10.3390/ijms26062465/s1.
