# Supplementary material for: Cartilage Oligomeric Matrix Protein Promotes Radiation Resistance in Non-Small Cell Lung Cancer In Vitro
Source: Int J Mol Sci. 2025 Mar 10;26(6):2465. doi: 10.3390/ijms26062465 (PMC11942305; doi:10.3390/ijms26062465)
Supplement: Supplementary file 1 [file ijms-26-02465-s001.zip › ijms-3441564-supplementary.pdf]

Supplementary Materials

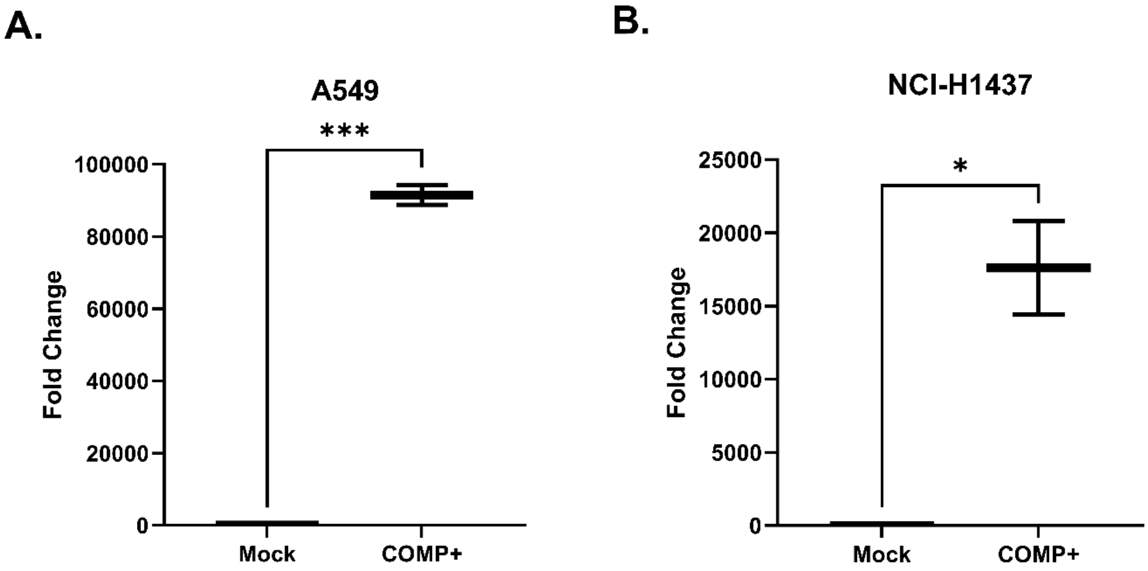

Figure S1. Q-PCR analysis of COMP mRNA expression in COMP-overexpressed and mock-transduced NSCLC cells. (A) COMP mRNA expression of mock-transduced and COMP-transduced A549 cells. (B) COMP mRNA expression of mock-transduced and COMP-transduced NCI-H1437 cells. Statistical analysis done by unpaired t-test. Error bars represent Min and Max (*p<0.05, ***p<0.001).

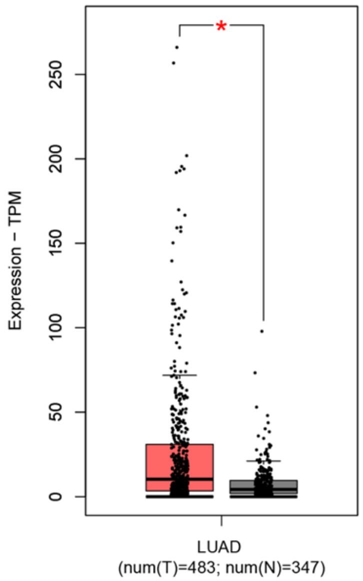

Figure S2. TCGA database analysis for COMP expression in lung adenocarcinoma. (A) RNA-seq data from the TCGA shows COMP expression is higher in lung adenocarcinoma (LUAD) tumor (T) tissue (n=483) compared to normal lung (N) tissue (n=347) (*p<0.05). Figure made using the GEPIA2 online tool [24].
